# Feasibility and Effectiveness of Basic Lymphedema Management in Leogane, Haiti, an Area Endemic for Bancroftian Filariasis

**DOI:** 10.1371/journal.pntd.0000668

**Published:** 2010-04-20

**Authors:** David G. Addiss, Jacky Louis-Charles, Jacquelin Roberts, Frederic LeConte, Joyanna M. Wendt, Marie Denise Milord, Patrick J. Lammie, Gerusa Dreyer

**Affiliations:** 1 Division of Parasitic Diseases, National Center for Infectious Diseases, U.S. Centers for Disease Control and Prevention, Atlanta, Georgia, United States of America; 2 Fetzer Institute, Kalamazoo, Michigan, United States of America; 3 Hôpital Ste. Croix, Leogane, Haiti; 4 Non-governmental Organization Amaury Coutinho, Recife, Brazil; Ghana Health Service, Ghana

## Abstract

**Background:**

Approximately 14 million persons living in areas endemic for lymphatic filariasis have lymphedema of the leg. Clinical studies indicate that repeated episodes of bacterial acute dermatolymphangioadenitis (ADLA) lead to progression of lymphedema and that basic lymphedema management, which emphasizes hygiene, skin care, exercise, and leg elevation, can reduce ADLA frequency. However, few studies have prospectively evaluated the effectiveness of basic lymphedema management or assessed the role of compressive bandaging for lymphedema in resource-poor settings.

**Methodology/Principal Findings:**

Between 1995 and 1998, we prospectively monitored ADLA incidence and leg volume in 175 persons with lymphedema of the leg who enrolled in a lymphedema clinic in Leogane, Haiti, an area endemic for *Wuchereria bancrofti*. During the first phase of the study, when a major focus of the program was to reduce leg volume using compression bandages, ADLA incidence was 1.56 episodes per person-year. After March 1997, when hygiene and skin care were systematically emphasized and bandaging discouraged, ADLA incidence decreased to 0.48 episodes per person-year (P<0.0001). ADLA incidence was significantly associated with leg volume, stage of lymphedema, illiteracy, and use of compression bandages. Leg volume decreased in 78% of patients; over the entire study period, this reduction was statistically significant only for legs with stage 2 lymphedema (P = 0.01).

**Conclusions/Significance:**

Basic lymphedema management, which emphasized hygiene and self-care, was associated with a 69% reduction in ADLA incidence. Use of compression bandages in this setting was associated with an increased risk of ADLA. Basic lymphedema management is feasible and effective in resource-limited areas that are endemic for lymphatic filariasis.

## Author Summary

Lymphatic filariasis is a parasitic disease that is spread by mosquitoes. In tropical countries where lymphatic filariasis occurs, approximately 14 million people suffer from chronic swelling of the leg, known as lymphedema. Repeated episodes of bacterial skin infection (acute attacks) cause lymphedema to progress to its disfiguring form, elephantiasis. To help achieve the goal of eliminating lymphatic filariasis globally, the World Health Organization recommends basic lymphedema management, which emphasizes hygiene, skin care, exercise, and leg elevation. Its effectiveness in reducing acute attack frequency, as well as the role of compressive bandaging, have not been adequately evaluated in filariasis-endemic areas. Between 1995 and 1998, we studied 175 people with lymphedema of the leg in Leogane, Haiti. During Phase I of the study, when compression bandaging was used to reduce leg volume, the average acute attack rate was 1.56 episodes per year; it was greater in people who were illiterate and those who used compression bandages. After March 1997, when hygiene and skin care were emphasized and bandaging discouraged, acute attack frequency significantly decreased to 0.48 episodes per year. This study highlights the effectiveness of hygiene and skin care, as well as limitations of compressive bandaging, in managing lymphedema in filariasis-endemic areas.

## Introduction

Lymphedema of the leg and its advanced form, known as elephantiasis, are major causes of disability and morbidity in filariasis-endemic areas, with an estimated 14 million cases worldwide [1]. When the World Health Organization's Global Program to Eliminate Lymphatic Filariasis (GPELF) was launched in 1998, its stated goals included not only interrupting transmission of the parasite, but also providing care to persons who suffer from clinical disease [2], [3]. During the 1990s, several studies in filariasis-endemic areas highlighted the importance of repeated episodes of acute bacterial dermatolymphangioadenitis (ADLA) in the progression of lymphedema severity [4]–[8]. These inflammatory episodes, characterized by intense pain, swelling, fever, and chills, accelerate damage to the peripheral lymphatic channels in the skin, which leads to worsened lymphatic dysfunction, fibrosis, and increased risk of further ADLA episodes. Clinical studies suggest that basic lymphedema management – including hygiene, skin care, elevation of the limb, and range-of-motion exercises – can halt, or perhaps even partially reverse, this progression [9]–[14]. The current prospective study was done to test, under field conditions, the feasibility and effectiveness of basic lymphedema management as a public health intervention in a resource-poor setting.

Leogane, Haiti, located approximately 30 km west of Port au Prince, has long been endemic for lymphatic filariasis; the prevalence of *Wuchereria bancrofti* microfilaremia was 16% in 2000 [15]. The outpatient clinic at Ste. Croix Hospital, the major health facility for Leogane Commune, was the site of this study. Lymphedema of the leg, which disproportionately affects women [16], is a major public health problem in Leogane [17]. As in many other areas where bancroftian filariasis is endemic, few persons with lymphedema of the leg remain infected with the parasite [18].

## Methods

The study was approved by the Ethics Committee of Ste. Croix Hospital and by the Institutional Review Board of the Centers for Disease Control and Prevention. Patients were included in the study if they had lymphedema of the leg, agreed to return to the hospital clinic for follow-up evaluations, had no obvious cause of edema such as tumor or congenital anomalies, and provided written informed consent. On their initial visit to the clinic, the volume of each leg was measured using a water displacement method, and each leg was classified as to stage of lymphedema using the 7-stage system of Dreyer and colleagues [19]. In this system, stage 1 is characterized by minimal swelling that reverses with horizontal rest. Stage 2 lymphedema does not completely reverse, but the skin appears normal. In stage 3 lymphedema the skin is thickened so that shallow skin folds are apparent. For purposes of analysis, we combined the Dreyer stages 4–7 into a single fourth-stage category; briefly, these are characterized by protrusions or “knobs” on the skin (stage 4), deep skin folds (stage 5), mossy lesions (stage 6) and severity that inhibits performing daily activities (stage 7) [19]. Lymphedema was considered bilateral if both legs had lymphedema of stage 2 or greater.

Information was collected on patient age, gender, literacy, education, duration of lymphedema, and number of ADLA episodes recalled by the patient during the previous 12 months. Patients were instructed how to wash their legs, apply antifungal and antibiotic creams or disinfectant (KMnO_4_) to the skin when indicated, elevate the foot of the bed, and perform range-of-motion exercises. They were asked to return to the hospital clinic every 4–6 weeks for routine follow-up, at which time information was collected on any ADLA episodes since the previous visit that had not been observed by clinic staff, as well as compliance with the components of basic lymphedema management. Compliance was assessed by asking each patient a standardized list of questions regarding the frequency of washing the leg, performing leg exercises, elevating the leg during the day, and sleeping with the foot of the bed raised since the previous visit. Frequency categories included “every day,” “most days,” “occasionally,” or “not at all.” Leg volume was again measured and patients were instructed and encouraged to continue practicing lymphedema management measures.

Patients also were told to return to the clinic if they developed signs of ADLA, where they were evaluated and given care, which included antibiotics, and, if necessary, hospitalized. This care was provided free of charge. Most patients provided their own supplies for washing the legs and feet, including soap, basins, and towels; these were provided free of charge to the few patients who did not already have and could not afford them. Antiseptic and topical antimicrobial agents for skin lesions were provided by the clinic free of charge to all patients. Other than free care and supplies, patients received no financial incentives to participate. Home visits were made on an ad-hoc basis to assess patient capacity for self-care and to encourage patient compliance. An effort also was made to conduct home visits when patients missed one or more regularly-scheduled clinic appointments. Data were not systematically recorded during these home visits.

### Pilot phase

In June 1995, the physical therapist at Ste. Croix (JLC) recruited a group of 30 patients with lymphedema of the leg to participate in a pilot project of basic lymphedema management. The goals of this pilot were to assess the feasibility and acceptance of basic lymphedema management in a small group of patients and to test the feasibility of volumetric measurement in this setting. During this pilot phase, leg volume sometimes was measured twice a day to assess diurnal variation (data not shown). The pilot was intended to last two months, but it continued with these 30 patients until the spring of 1996, when increased funding allowed the project to scale up. For the purposes of analysis, the pilot study is considered as part of Phase I.

### Phase I

With the project expansion in March 1996, eight additional staff, including a nurse, a community health worker, and others with high school-level education but no health background were trained as lymphedema technicians. A hospital physician (FL) provided consultation and medical care as needed. During Phase I, an attempt was made to compare the effectiveness of basic lymphedema management with a more intensive method that included compressive bandaging, a component of “complex decongestive physiotherapy” [20]. Two to four weeks of compressive bandaging (using Comprilan®) resulted in rapid volume reduction, which was maintained with a locally designed and produced compressive garment made of Velcro®. However, because it resulted in rapid volume reduction, the popularity of compressive bandaging made it impossible to randomize patients to the non-bandage intervention group.

### Phase II

In March 1997, Dr. Gerusa Dreyer, who had developed a basic lymphedema management program in Recife, Brazil, visited Leogane to review the project and to conduct training for the staff. As a result of that visit, the program shifted its primary focus from reducing leg volume to preventing ADLA through hygiene and skin care. Use of compressive bandaging and garments was no longer encouraged and their use declined dramatically. In November 1997, a colorful booklet with the messages of basic lymphedema management was given to each patient, and a “soap opera” was broadcast over local radio that depicted the life of a young woman with lymphedema and how she benefited from lymphedema self-care.

### Statistical analysis

The analysis was limited to patients who returned to the clinic for at least 5 routinely scheduled visits over a period of least 6 months. We considered shorter periods of observation inadequate to evaluate the effect of the intervention. ADLA incidence was calculated as episodes per person-year of observation. Changes in leg volume were calculated by subtracting last-measured volume from initial volume during a specified time period. The pooled t-test was used to compare differences in age by study phase; the paired t-test to compare changes in leg volumes; the chi-square or Fisher's Exact test to compare proportions associated with gender and literacy; and Poisson regression to calculate and compare ADLA incidence rates and to identify factors associated with ADLA episodes. The effect of factors associated with leg volume was investigated using multivariate regression. All regressions were run using SAS proc genmod, using the generalized estimating equations (GEE) procedure to adjust for correlation of multiple observations (e.g., ADLA episodes in different legs) from the same individual over time [21]. Analyses were performed using SAS version 9.2, SAS Institute Inc, Cary, NC, USA. Statistical significance was set at alpha = 0.05.

## Results

A total of 302 patients first received lymphedema care at the Ste. Croix Hospital clinic between June 1995 and December 1997. They came from filariasis-endemic areas throughout the country. Clinic resources were sufficient during this time to enroll 230 patients who met the inclusion criteria. Of these, 175 (76%) returned to the clinic for at least 5 routinely scheduled visits over a period of least 6 months. A total of 145 (82.9%) of these patients were female and the mean age was 37.3 years (range, 10–85). Eighty-four percent lived within Leogane Commune. Eighty-seven (49.7%) patients reported they could read and write.

Reported duration of edema at the time of entry into the study was 11.3 years (range, <1 to 50) (Table 1). Using the 4-stage classification of lymphedema, 78 (44.6%) patients had skin changes characteristic of stage 3 and 12 (6.9%) had frank elephantiasis, stage 4. Lymphedema stage was not significantly associated with gender (P = 0.76). Fifty-five (31.4%) patients had bilateral lymphedema (≥stage 2). Mean volume of affected legs on entry into the study ranged from 1586 mL for unaffected legs to 3422 mL for stage 4. Volume increased significantly with lymphedema stage (P<0.001), although considerable variation was observed within each stage.

**Table 1:** Characteristics of 175 patients with lymphedema who participated in the study, including 127 persons who entered before April 1, 1997 (during Phase I) and 48 who entered after April 1, 1997 (Phase II), univariate analysis.

Characteristic	All patients	Entered in Phase I (before April 1997)	Entered in Phase II (after April 1, 1997)	P*
No. persons	175	127	48	
Mean age in years (range)	37.3 (10–85)	36.6 (10–85)	39.2 (12–75)	0.34
No. (%) female	145 (82.9%)	108 (85.0%)	37 (77.1%)	0.26
No. (%) literate	87 (49.7%)	58 (45.7%)	29 (60.4%)	0.09
Mean (range) duration of lymphedema upon entry into study (years)	11.3 (<1–50)	10.5 (<1–50)	13.3 (<1–38)	0.10
Mean no. of reported ADLA episodes in the year before entering the study (range)	2.1 (0–13)	2.4 (0–13)	1.3 (0–6)	0.0004
No. legs	350	254	96	
Lymphedema stage				
0 (no edema)	85 (24.3)	59 (23.2)	26 (27.1)	0.1467
1	38 (10.8)	29 (11.4)	9 (9.4)	
2	129 (36.9)	88 (34.7)	41 (42.7)	
3	85 (24.3)	70 (27.6)	15 (15.6)	
4	13 (3.7)	8 (3.1)	5 (5.2)	

* Comparing 127 persons entering the study in Phase I with the 48 who entered in Phase II.

The 175 patients were observed for a total of 3874 months, for a mean of 22.1 months (range, 6.3–41.2). The number of routine clinic visits ranged from 5 to 123 (mean, 26.9). In all, 127 patients entered the study during Phase I (July 1995–March 1997) and an additional 48 entered during Phase II (after April 1, 1997). These two groups did not differ significantly by age, sex, literacy, or lymphedema stage or duration, but patients who entered in Phase II reported having had significantly fewer ADLA episodes during the 12 months before entering the study (Table 1). All patients continued through the end of the study in December 1998.

### ADLA

During the 12 months before entering the program, the 175 patients reported a mean of 2.1 episodes of ADLA (range 0–13), with a mean duration of 2.6 days each (range <1 day–44 days). Reported ADLA incidence by 12-month recall was not associated with patient age or sex but was significantly associated with illiteracy (RR 1.6, P = 0.01), bilateral lymphedema (RR 1.6, P = 0.02) and greater lymphedema stage (0.67, 1.72, 2.32, and 3.08 episodes per year for persons with stages 1 through 4, respectively, P = 0.01).

During the study, a total of 242 ADLA episodes were reported, for an overall incidence of 0.75 episodes per person-year, with a range of 0 to 10 per patient. Of these episodes, 141 (58%) were witnessed by clinic staff. Mean reported duration of each ADLA episode was 3.9 days (range 1–22). Eighty-seven patients (49.7%) had no ADLA during the study, 67 (38.3%) had an annual incidence of <2.0 per person-year, and 21 (12.0%) experienced two or more episodes per year. In univariate analysis, ADLA incidence during the study was positively associated with lymphedema stage and leg volume, illiteracy, use of compression bandages or garments, and reported frequency of ADLA before entering the study (Table 2). No consistent seasonal pattern was observed for ADLA incidence, although it tended to increase between the third and fourth quarters of the year; this increase was statistically significant (P = 0.006) only for 1997.

**Table 2:** Incidence of acute dermatolymphangioadenitis (ADLA), in episodes per person-year, among 175 patients with lymphedema in Leogane, Haiti, by demographic, disease-related, and treatment-related factors, univariate analysis.

Characteristic	Value or status	N	ADLA incidence (range)	P
Gender	Male	30	.83 (0–5.6)	.4946
	Female	145	.74 (0–2.9)	
Age (years)	≥40	80	.77 (0–5.6)	.8093
	<40	90	.75 (0–3.2)	
Literacy	Illiterate	88	1.01 (0–5.6)	<.0001
	Literate	87	.47 (0–2.5)	
Bilateral lymphedema (>stage 2 bilaterally)	Yes	55	.87 (0–3.0)	.0740
	No	120	.69 (0–5.6)	
Lymphedema duration (years)	≥10	78	.72 (0–3.8)	.6275
	<10	97	.77 (0–5.6)	
Median leg volume (n = 350 legs)	≥2000 mL	125	1.35	<.0001
	1700–1999 mL	117	.52	.13
	<1700 mL	108	.33	Referent
ADLA frequency during the year before entering the study, based on 12-month recall (episodes per year)	>2.0	84	.94 (0–5.6)	.0004
	0.1–2.0	55	.59 (0–3.8)	.2416
	0	36	.44 (0–2.9)	Referent
Ever used compression bandages or garments during study	Yes	122	.88	<.0001
	No	53	.43	Referent
Lymphedema stage, by leg (n = 350 legs)	4	13	1.08	<.0001
	3	85	1.52	<.0001
	2	129	.76	<.0001
	1	38	.37	.0015
	0	85	0	Referent

**Figure 1:**
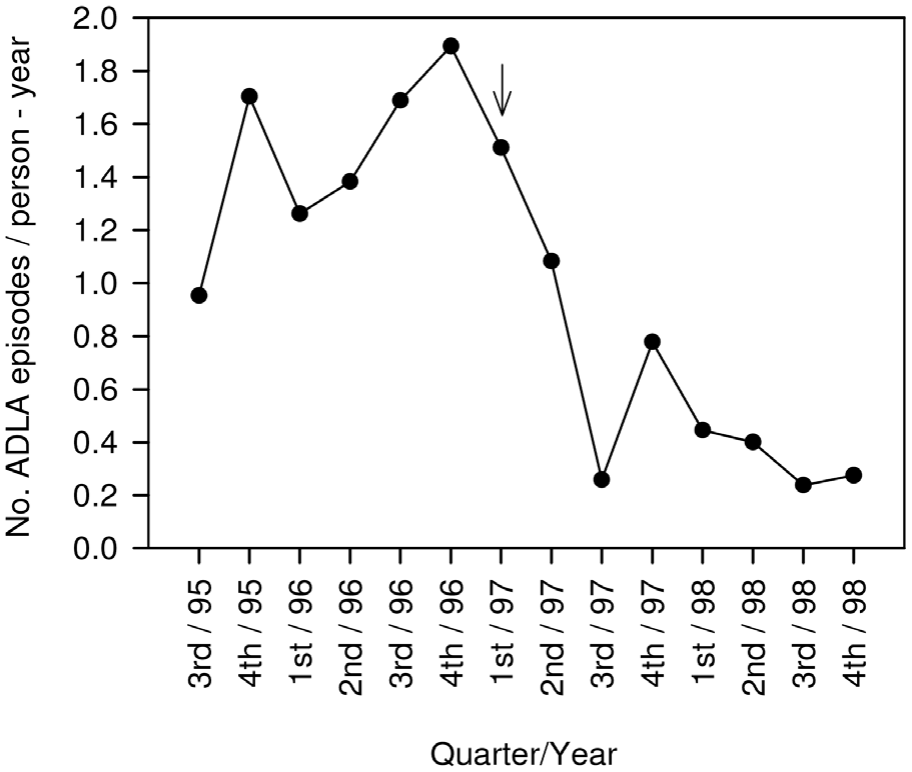
Quarterly incidence of acute dermatolymphangioadenitis (ADLA) in 175 lymphedema patients, 1995–1988, Leogane, Haiti. Arrow indicates the point at which the program focus shifted away from decreasing leg volume using compression bandages to reducing the frequency of ADLA through hygiene, skin care, range-of-motion exercises and leg elevation.

Because the emphasis of the program shifted from leg volume reduction during Phase I to ADLA prevention through self-care during Phase II, we compared ADLA incidence for these two periods (Figure 1). In Phase I, the incidence of ADLA was 1.56 episodes per person-year, compared to 0.48 overall in Phase II (P<0.0001). A significant reduction was observed even when the analysis was restricted to those who entered the study during Phase I. Among these 127 persons, ADLA incidence decreased to 0.54 per person-year during Phase II (P<0.0001). ADLA incidence during Phase II was even lower for the 48 persons who entered the study during Phase II, 0.22 episodes per person-year (P<0.0001) (Table 3).

**Table 3:** Acute dermatolymphangioadenitis (ADLA) among the 127 lymphedema patients who entered the study during Phase I (before April 1, 1997); for the 48 who entered the study during Phase II (after April 1, 1997); and for all 175 participants combined, univariate analysis.

Characteristic	Patients Who Entered the Study During Phase I (N = 127)				Patients Who Entered the Study During Phase II (N = 48)		All Participants (N = 175)	
Period of Observation	Year before Study*	Phase I	Phase II	Phases I & II	Year before Study*	Phase II	Year before Study*	Phases I & II
No. person-years of follow-up	127	81.6	191.8	273.4	48	49.3	175	322.8
No. ADLA episodes	298	127	104	231	63	11	361	242
ADLA incidence**	2.35	1.56†	0.54† ‡	0.84	1.31	0.22‡	2.06	0.75
No. (%) persons with ADLA incidence of:								
0	24 (18.9%)	71 (55.9%)	68 (53.5%)	47 (37.0%)	12 (25.0%)	40 (83.3%)	36 (20.6%)	87 (49.7%)
0.1–1.99 per year	35 (27.6%)	21 (16.5%)	47 (37.0%)	61 (48.0%)	20 (41.7%)	6 (12.5%)	55 (31.4%)	67 (38.3%)
≥2.00 per year	68 (53.5%)	35 (27.6%)	12 (9.5%)	19 (15.0%)	16 (33.3%)	2 (4.2%)	84 (48.0%)	21 (12.0%)
ADLA incidence**								
Any use of compression bandage or garment	–	1.76	0.52	0.97	–	0.25	–	.88
No use of compression bandage or garment	–	1.00	0.57	0.50	–	0.19	–	.43
P value¶		<.0001	0.51	.0002		.68		.0001

* Based on 1-year recall.

** ADLA episodes per person-year.

† P<0.0001, ADLA incidence in Phase I vs. Phase II for persons participating during both phases.

‡ P<0.0001, ADLA incidence in Phase II, comparing persons who entered during Phase I and those who entered in Phase II.

¶ Comparing ADLA incidence for persons who used compression bandages or garments and those who did not.

A multivariate Poisson regression analysis considered the effect of treatment compliance, illiteracy, gender, and use of compression bandages on ADLA incidence among the 127 persons who entered the study during Phase I, when compressive bandaging use was most common. Only illiteracy and use of compression bandages or garments were significantly associated with ADLA incidence. A significant (P = 0.02) interactive effect of bandaging and illiteracy was observed. Among illiterate persons, mean ADLA incidence did not change with or without use of compression bandages (1.1 episodes per year vs. 1.0 episode per year, respectively). However, among literate persons, those who ever used compression bandages had an overall ADLA incidence of 0.76 per year, compared to 0.20 per year for those who never used compression bandages or garments (P = 0.005).

### Leg volume

Of 175 patients, 137 (78.3%) experienced some reduction in leg volume during the study; volume decreased in 66.4% of lymphedematous legs (median reduction, 90 ml). In general, volume decreased dramatically following application of compressive bandages and increased rapidly during ADLA. Compared to leg volume on entering the study, mean leg volume at the end of the study was 14 mL greater in normal legs and 5 mL greater in legs with stage 1 lymphedema. Mean decreases of 59 mL, 93 mL, and 571 mL were observed for lymphedema stages 2, 3, and 4, respectively, statistically significant over the entire study period only for stage 2 (P = 0.01) (Table 4). Controlling for study phase, volume reduction in all 175 patients was significantly associated with increasing lymphedema stage (P = 0.005), lower frequency of ADLA during the study period (P = 0.016), and use of compressive bandages (a mean decrease of 86.6 mL, compared to 8.2 mL in lymphedematous legs in which no compression was used, P = 0.039), but not with age or sex.

**Table 4:** Mean leg volume, in mL, among lymphedema patients in Leogane, Haiti, a) upon study entry, b) at the beginning of Phase II, and c) at the end of the study, by lymphedema stage and phase during which the patient entered the study.

Stage	Entered study during Phase I (N = 127 persons, 254 legs)				Entered study during Phase II (N = 48 persons, 96 legs)			All participants (N = 175 persons, 350 legs)		
	No. legs	Mean pre-treatment leg volume at beginning of Phase I (range)	Mean leg volume at end of Phase I (beginning of Phase II) (range)	Mean leg volume at end of Phase II (range)	No. legs	Mean pre-treatment leg volume at the beginning of Phase II (range)	Mean leg volume at end of Phase II (range)	No. legs	Mean leg volume on entry into the study (range)*	Mean leg volume on last date of participation in the study
0	59	1573 (1110–2445)	1592 (1195–2460)	1598 (1145–2360)	26	1610 (1080–2160)	1604 (1310–2130)	85	1586 (1080–2445)	1600 (1145–2360)
1	29	1664 (1205–2155)	1714^1^ (1360–2215)	1718 (1250–2285)	9	1937 (1515–2760)	1786 (1470–2030)	38	1729 (1205–2760)	1734 (1250–2285)
2	88	1994 (1020–3220)	1953^1^ (1460–2890)	1947^1^ (1260–2945)	41	1986 (1450–2835)	1909^2^ (1230–2970)	129	1991 (1020–3220)	1935^1^ (1230–2970)
3	70	2328 (1225–4540)	2293 (1645–3720)	2317 (1645–3910)	15	2839 (1920–3700)	2354^2^ (1850–3260)	85	2417 (1225–4540)	2324 (1645–3910)
4	8	3284 (2515–4595)	2903^1^ (2135–3750)	2706^1^ (1820–3500)	5	3644 (2390–4760)	3082 (1510–4010)	13	3422 (2390–4760)	2851^3^ (1510–4010)

1 Statistically significant (P = 0.01) difference compared to mean leg volume at entry into the study.

2 Statistically significant (P<0.05) difference compared to mean leg volume at the beginning of Phase II.

3 P = 0.05, compared to mean leg volume at entry into study.

* Regardless of which phase the patient entered the study.

### Reported compliance

For the purposes of data analysis, compliance with the four recommended practices of leg washing, exercises, leg elevation, and sleeping with the foot of the bed raised was defined as having reported, on at least 75% of all clinic visits, that the behaviors had been practiced every day since the previous clinic visit. Using this definition, compliance rates were 88.0%, 38.3%, 69.7%, and 49.7% for these four practices, respectively. Significant increases in daily compliance between Phase I and Phase II were observed for leg washing (from 61.7% to 94.3%, P<0.001), leg elevation (59.2% to 68.6%, P = 0.01), and sleeping with the foot of the bed elevated (40.8% to 51.4%, P<0.001). Overall compliance was calculated by summing the percentage figures for all four recommended practices; a score of ≥300 (of a possible 400) was considered compliant. Overall compliance increased from 45.8% to 66.3% between Phase I and Phase II (P = 0.003). In a multivariate Poisson regression analysis, compliance was significantly associated with female gender (OR 1.6, 95% Confidence Interval [CI] 1.0–2.5, P = 0.05) and age >40 years (OR 1.5, 95% CI 1.1–2.0, P = 0.02), but not with literacy or lymphedema stage.

## Discussion

This prospective study provides evidence that basic lymphedema management is feasible and acceptable to patients in resource-poor areas where lymphatic filariasis is endemic. In general, patients were able to incorporate basic self-care measures, especially leg washing, into their daily routines. When proper hygiene and skin care were emphasized, the incidence of ADLA decreased rapidly to 31% of earlier levels and these reductions were sustained over time. A follow-up study by Dahl and colleagues in 2000–2001 showed that ADLA incidence in these patients remained low and even decreased further (Dahl, BA, unpublished thesis, Emory University, Atlanta, Georgia).

Recent studies indicate that, in addition to being a critical risk factor for progression of lymphedema [4]–[8], [13], [14], ADLA episodes are strongly associated with poor quality of life [10], [22]–[28]. Thus, reducing ADLA frequency is arguably the most important objective of lymphedema management in resource-poor countries. Accordingly, ADLA has emerged as a key indicator for monitoring lymphedema management programs in filariasis-endemic areas [29].

In contrast, the usefulness of leg volume as an indicator of clinical improvement in filariasis-endemic areas seems limited. Leg volume, albeit an “objective” measure, varied widely with use of compression garments and, to an extent, with time of day (data not shown). Overall, leg volume decreased in 78% of our patients, but these reductions were generally modest, and statistically significant only for stage 2 lymphedema. Our initial focus on reducing leg volume, although popular with patients, distracted attention from hygiene and skin care; as a result, no significant reduction in ADLA incidence was observed during Phase I of the study. Compression bandages reduced leg volume quickly, but their use was associated with an increase in ADLA incidence. It was difficult to keep the compression bandages clean, and they were prohibitively expensive – equivalent to the annual income of many patients. Frequent visits to the clinic for reapplication of compression bandages in the initial stages of treatment also fostered dependence on clinic staff and undermined key messages of self-reliance and self-care. Thus, although reduction in leg volume is a desirable outcome, our experience suggests that it should not be the primary goal of lymphedema treatment in resource-poor settings.

After receiving training from Dr. Dreyer midway through the study, the clinic staff was better equipped to deliver simple, clear, assertive messages to patients regarding hygiene and skin care. These messages were later reinforced by a simple booklet that was given to each patient, opportunities to participate in support groups, and a radio drama about a young woman with lymphedema that was aired repeatedly on local radio. With these interventions, ADLA incidence dropped dramatically and remained low. The relative contribution of each component of the overall intervention is unclear; we believe that they acted synergistically. Coreil and colleagues showed that patients in Leogane who regularly participated in support groups had a lower incidence of ADLA than those who did not [30]. In addition, self-efficacy (belief in one's ability to perform lymphedema self-care) increased significantly following distribution of booklets and broadcasts of the radio dramas (Wendt, JM, unpublished thesis, Emory University, Atlanta, GA).

The number and duration of patient contacts required for patients to understand, become proficient in, and fully committed to life-long lymphedema self-care is not clear. In practice, the intensity of patient training and follow-up varies among filariasis programs [29]. Our results indicate that patients who were illiterate and those with advanced disease continued to be at risk of ADLA episodes. These results suggest that more intensive effort may be needed for such patients, such as home visits and support groups. The fact that illiterate persons remained at a 2-fold risk of ADLA throughout the study, even when controlling for other factors, also suggests that illiteracy may be a marker for other factors related to ADLA incidence, such as lower self-efficacy or increased risk of skin lesions that predispose to ADLA [31], [32].

In addition to reduced ADLA incidence, we observed other improvements in quality of life among patients in this study. These were often dramatic. Patients commonly reported decreased stigma and shame and a return to active life, including school or work [30, Wendt JM, unpublished]. Those with advanced lymphedema and multiple skin folds and lesions reported, often for the first time in years, the elimination of offensive odor. Serial biopsies in a subset of patients confirmed a decrease in chronic inflammation in lymphedematous legs [33].

Despite these remarkable changes at the tissue level, most patients did not experience reduction in lymphedema stage. The 4-stage classification that we used is not particularly sensitive to localized changes, and considerable heterogeneity can be found within each stage. Most of the lymphedema classification and staging systems that have been proposed suffer from these limitations [34].

Studies have reported that the incidence of adenolymphangitis (presumably ADLA or ADLA-like) may decrease following mass treatment with antifilarial drugs [35]–[38]. The current study was completed almost two years before mass treatment was initiated in Leogane [15]; diethylcarbamazine was not readily available. Therefore, it seems unlikely that the clinical improvement and decreased ADLA frequency that we observed can be attributed to reduced transmission of *W. bancrofti*.

Seasonal fluctuations in ADLA, associated with rainfall, have been reported in other studies [39]. We observed no consistent seasonal trends in ADLA incidence. The tendency for ADLA incidence to increase between the third and fourth quarters was statistically significant only for 1997. The reason for this is not clear. Rainfall patterns varied from year to year in Haiti during the study period, and the fall of 1997 was relatively dry.

Prospective ADLA monitoring, as was done in this study, is accurate, but expensive. The accuracy of patient recall over longer periods is unknown, but the pain and suffering associated with ADLA make it a memorable event. Among the 127 patients who entered the study during Phase I, the observed incidence of ADLA during Phase I was 1.6 episodes per person-year, compared to 2.4 self-reported for the 12 months before entering the study. It is risky to compare retrospective and prospective data, and it also is possible that messages regarding hygiene and skin care during Phase I, albeit sub-optimal, might have reduced ADLA incidence from baseline levels. However, the data suggest that 12-month recall may be adequate for rapid epidemiologic assessments or program monitoring. Additional research is warranted to assess reliability of 12-month ADLA recall in different filariasis-endemic areas.

The preponderance of women in our study sample reflects the gender distribution among persons with lymphedema in the Leogane population [16]. In many areas where bancroftian filariasis is endemic, lymphedema of the leg is more common in women than in men, although this finding is not universal [38].

This study has several limitations. First, both the incidence of ADLA and compliance with lymphedema self-care were assessed by self-report – a method that, typically, is less than completely reliable. Recall of ADLA episodes during the year before entering the study was facilitated by careful questioning and using memorable public events to help “frame” the previous 12 months. During the study, patients were encouraged to report ADLA and compliance accurately and honestly, and they were assured that clinical care would continue, free of charge, regardless of what they reported. Some 58% of ADLA episodes reported by patients were observed and confirmed by research staff at the clinic. In many of the remaining cases, patients were seen within 1–2 weeks after the episode, when clinical signs (such as peeling of the skin, warmth, or edema [19]) were still visible. Skin condition and interdigital lesions were carefully assessed during routine clinic visits, and they provided a check on self-reported compliance. Ad-hoc home visits also encouraged accurate self-reporting. For example, the absence of soap, a clean towel, or an appropriate washing basin would call into question high levels of self-reported compliance.

Second, because of ethical concerns about withholding treatment, we did not collect comparative prospective data on a control group of patients. Further, although we desired to randomize patients with regard to compressive bandaging, we were unable to do so. However, we were able to compare ADLA incidence as a function of the focus of our programmatic intervention (i.e., leg volume during Phase I and ADLA incidence during Phase II).

Third, because of the conditions under which this study was conducted, it cannot be regarded as a study of the efficacy of lymphedema management. We had limited on-site access to specialists in lymphedema management, our staff was comprised mostly of motivated young people rather than health professionals, and the intervention evolved as we gained experience. It is likely that even greater reductions in ADLA frequency would be observed under more optimal conditions.

Despite these limitations, our results suggest that basic lymphedema management is both feasible and effective in filariasis-endemic areas where resources are limited. When provided with the skills and motivation to practice lymphedema self-care, most patients will do so. However, simple messages that unequivocally emphasize the importance of hygiene and skin care are important. Our experience also suggests that compressive bandages, although useful for individual patients, should not be the mainstay of treatment in filariasis-endemic areas, and that volume reduction may not be the optimal measure of success.

## Supporting Information

Alternative Language Abstract S1 Translation of the abstract into French by MDM.
(0.03 MB DOC)
