# Supplementary material for: Feasibility and Effectiveness of Basic Lymphedema Management in Leogane, Haiti, an Area Endemic for Bancroftian Filariasis
Source: PLoS Negl Trop Dis. 2010 Apr 20;4(4):e668. doi: 10.1371/journal.pntd.0000668 (PMC2857874; doi:10.1371/journal.pntd.0000668)
Supplement: Alternative Language Abstract S1 — Translation of the abstract into French by MDM. (0.03 MB DOC) [file pntd.0000668.s001.doc]

**Faisabilite et efficacite de la prise en charge de base du lymphoedeme a Leogane, Haiti, une zone ou la filariose bancroftienne est endemique**

*Background*-Approximativement 14 million de personnes vivant dans les zones ou la filariose lymphatique est endemique presentent un lymphoedème du membre inférieur. Les études cliniques démontrent que les episodes fréquents d’adénodermatolymphangites ( ADLA ) entraînent la progression du lymphoedème et que la prise en charge de base du lymphoedème, laquelle est basée sur l’hygiène, les soins de la peau, les exercices et la surélévation du membre, peut réduire la fréquence de l’ADLA. Cependant, peu d’études ont évalué l’efficacité de la prise en charge de base du lymphoedème de façon prospective ou ont évalué le role des bandages compressifs pour les cas de lymphoedème dans les endroits ou les resources sont manquantes.

*Methodologie / Principaux resultat*s. Entre 1995 et 1998, nous avons monitoré de facon prospective l’ADLA et le volume du membre inférieur chez 175 personnes avec lymphoedème de la jambe, enrollés dans une clinique de lymphoedème à Léogane, Haiti, une zone où le Wuchereria bancrofti est endémique. Durant la première phase de l’étude , alors que le programme portait l’emphase sur la réduction du volume de la jambe en utilisant les bandages compressifs, l’incidence de l’ADLA était de 1.56 épisodes par personne par année. L’incidence de l’ADLA était grandement associée avec le volume de la jambe, le stade de lymphoedème, l’analphabétisme, et l’usage de bandages compressifs. Après Mars 1997, quand l’emphase fut portée systematiquerment sur l’hygiène et les soins de la peau, et que l’usage des bandages compressifs fut découragé, l’incidence d’ADLA diminua de 0.48 épisodes par personne par année ( P <0.0001 ) Le volume de la jambe diminua chez 78% des patients

*Conclusion / Importance* La prise en charge de base du lymphoedème, laquelle porta l’emphase sur l’hygiène et les soins personnels était associée à 69 % de reduction dans l’incidence d’ADLA. Cependant, l’usage de bandages compressifs dans cet endroit était associé à un risqué incrémenté de ADLA. La prise en charge de base du lymphoedème est faisable et efficace dans les pays pauvres où la filariose lymphatique est endémique.
